# Effect of Climate Change on the Potentially Suitable Distribution Pattern of *Castanopsis hystrix* Miq. in China

**DOI:** 10.3390/plants12040717

**Published:** 2023-02-06

**Authors:** Linlin Shen, Haiyan Deng, Ganglong Zhang, Anqi Ma, Xiaoyong Mo

**Affiliations:** 1 College of Forestry and Landscape Architecture, South China Agricultural University, Guangzhou 510642, China; 2 Guangzhou Institute of Forestry and Landscape Architecture, Guangzhou 510405, China

**Keywords:** MaxEnt, ArcGIS, climate change, *Castanopsis hystrix* Miq., potentially suitable distribution pattern, present and future, SSP

## Abstract

Climate warming poses a great threat to ecosystems worldwide, which significantly affects the geographical distribution and suitable growth area of species. Taking *Castanopsis hystrix* Miq. as the research object, the potentially suitable cultivation regions under present and future climatic emission scenarios in China were predicted based on the MaxEnt model with 360 effective individual distributions and eight environmental variables. The min temperature of coldest month (bio6), precipitation of driest month (bio14), and precipitation of warmest quarter (bio18) are three leading factors affecting the geographical distribution area of *C. hystrix* Miq. The suitable cultivation regions of *C. hystrix* Miq. range from 18°–34° N, 89°–122° E in central and southern China and cover an area of 261.95 × 10^4^ km^2^. The spatial pattern of *C. hystrix* Miq. will migrate to the southern region of low latitudes with a decreasing suitable area when in ssp1-2.6, and to the southwestern region of low latitudes or expand to the northeast region at high latitudes in ssp5-8.5, with an increasing suitable area; no significant change on the spatial pattern in ssp2-2.4. For ssp1-2.6 or ssp2-4.5 climate scenarios, the southern region of high latitudes will be appropriate for introducing and cultivating *C. hystrix* Miq., and the cultivation area will increase. For ssp5-8.5, its cultivation will increase and expand to the northeast of high-latitude areas slightly.

## 1. Introduction

In its fifth assessment report, the United Nations Intergovernmental Panel on Climate Change (IPCC) stated that global warming has become a significant problem [1,2]. The most recent sixth assessment report on climate change in 2021 shows that climate change will continue for a short period, and it is anticipated that the global average surface temperature will rise by 1.5 °C in the next 20 years [3]. Dramatic changes in climate and the environment pose a great threat to the ecosystems of all regions of the world. At the macroscale, the geographical and spatial distribution of species is closely related to changes in climatic conditions and the natural environment [2,4,5,6,7]. Research shows that under climate change, especially with an increase in temperature and changes in precipitation, some plants would adapt by changing their own characteristics, resulting in changes in species composition, spatial distribution, and migration laws, leading to further damage to physiological characteristics and ecosystem functions [4,8,9,10]. Data have shown that because of a rise in temperature, the distribution of many tree species tends to migrate and expand to high altitudes or even polar regions [2,11,12,13,14,15]. Additionally, climate change may lead to the migration of some tree species to low altitudes [16]. Therefore, it is of great significance to study the potentially suitable cultivation regions for different species under climate change to conserve resources and stabilize ecosystems.

With the development of statistical modeling and geographic information systems (GIS), species distribution models (SDMs) have been widely used to evaluate the correlation between species sample information and environmental characteristics, including genetic algorithms for rule set prediction (GARP), ecological niche factor analysis (ENFA), maximum entropy models (MaxEnt), domain models (DOMAIN), and climate change experiments (CLIMEX), among other models. The MaxEnt model was first proposed by Phillips et al. [17]. It is a species geographic-scale spatial distribution model based on the principle of maximum entropy [18]. The theory of maximum entropy states that under known conditions, objects are closest to their true state when entropy is at a maximum [19]. The operational principle of the MaxEnt model is that the optimal estimation of the unknown distribution based on the known sample information should meet the known restrictions on the unknown distribution and make the distribution have the maximum entropy, to predict the habitat suitability distribution and climate suitability of the target species in the study area and present it in the form of a graph [19]. According to research, the MaxEnt model has a more stable operation, faster operation speed, and higher prediction accuracy than other models, and can also obtain better prediction results when there is a lack of species distribution points [17,20,21,22]. At present, many scholars have used the advantages of MaxEnt model to carry out a lot of research. Huang et al. [8] used the MaxEnt model to predict the potential area distribution of Toona ciliata var. pubescens and pointed out that the global potential area distribution pattern of Toona ciliata var. pubescens was continuous on the whole, but patchy in the high distribution area. Wang et al. [19] studied the climatic ecological suitability and potential distribution of Tricholoma matsutake on the Western Sichuan Plateau using the MaxEnt model and reported that the suitable areas of T. matsutake on the Western Sichuan Plateau were distributed in the southwest, south, middle, and east of the plateau at an altitude of 1900–3600 m. Sharma et al. [23] used a MaxEnt model to study the suitable habitat of Perilla frutescens in Uttarakhand and reported that the areas highly suitable for P. frutescens cultivation are the Dehradun, Tehri Garhwal, Uttarkashi, Rudraprayag, and Nainital districts of Uttarakhand, India.

*C. hystrix* Miq. is an excellent local timber tree, which has the characteristics of fast growth, good wood quality, and strong adaptability [24,25]. It is mainly distributed in eastern Fujian, southwestern Hunan, Guangdong, Hainan, Guangxi, Guizhou, and other areas in China [25]. As a national class II protected rare broad-leaved tree species with rapid growth in tropical and subtropical areas of China, it is often used as a mixed afforestation tree species in ecological public welfare forests to fully maximize its ecological benefits. Scholars and experts have conducted many studies on carbon storage, carbon sequestration characteristics, and distribution, which have confirmed that *C. hystrix* Miq. has good carbon sequestration capacity [19,26,27]. Climate change is closely related to the carbon cycle. The construction of pure and mixed forests of *C. hystrix* Miq. will help stabilize and even reduce the concentration of greenhouse gases in the atmosphere and slow the process of climate warming.

Nonetheless, in recent years, with global climate change, the distribution of *C. hystrix* Miq. has been gradually reduced and fragmented; coupled with unscientific cultivation management, unreasonable development and utilization, the survival and development of *C. hystrix* Miq. populations are highly threatened. Planting *C. hystrix* Miq. in China in the 1960s mainly focused on mixed experimental plantations [8], cultivation techniques [25], ecological benefits [8], and growth law [28], among others. There are few reports predicting the habitat suitability based on the SDM. Therefore, the potentially suitable cultivation regions for *C. hystrix* Miq. were simulated using the MaxEnt model and ArcGIS software under the present climatic conditions and three different emission scenarios for the years 2040 and 2060. We attempted to determine: (1) how climate and geographic factors affect the potentially suitable cultivation regions of *C. hystrix* Miq. and the dominant factors restricting its distribution; (2) division of potentially suitable cultivation regions for *C. hystrix* Miq. for the different suitability values; and (3) potentially suitable cultivation regions for *C. hystrix* Miq. in response to climate change during different periods. The solution to these problems can not only help us understand the biogeography of *C. hystrix* Miq. and implement conservation strategies to minimize the impacts of climate change but also provide a theoretical basis for effectively exerting the ecological functions of *C. hystrix* Miq. plantations, such as by regulating climate and enhancing carbon sequestration. These steps help to provide a scientific basis for the follow-up *C. hystrix* Miq germplasm resource protection, promotion and planting, forest transformation, ecological restoration and other issues.

## 2. Results

### 2.1. Model Prediction Accuracy Evaluation

**Figure 1:**
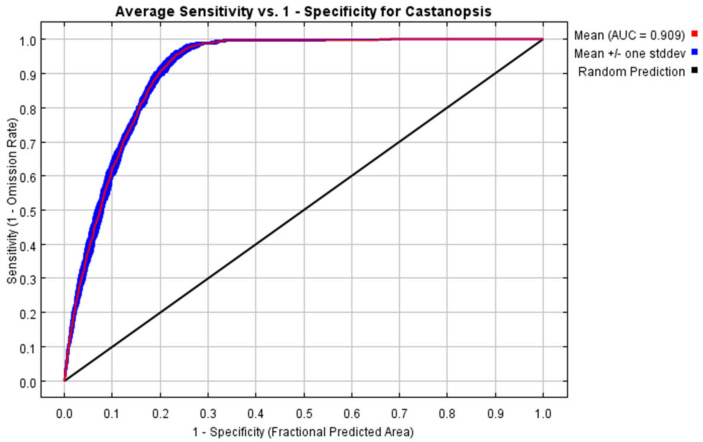
The subject operating characteristic curve generated by MaxEnt model.

Figure 1 shows the ROC curve evaluation results of the MaxEnt model after repeated operation ten times under the present climatic conditions, and the AUC value is the average value of the ten runs. The average AUC value and average standard deviation of the training set were 0.909 and 0.004, respectively, indicating that the MaxEnt model has an excellent simulation effect on the potentially suitable cultivation regions of *C. hystrix* Miq., and the result is highly reliable.

### 2.2. Importance Assessment of Environmental Variables

The results of the percent contribution and permutation importance evaluation method, which assesses the relative importance of the eight environmental variables in predicting the potential distribution of *C. hystrix* Miq. under the current climatic conditions, are listed in Table 1. Precipitation of driest month (bio14) and min temperature of coldest month (bio6) contributed 55.4% and 31.9% to the simulation of *C. hystrix* Miq. distribution, respectively, which were much higher than those of the other six environmental variables. The permutation importance of the min temperature of coldest month (bio6) and precipitation of warmest quarter (bio18) were 61.9% and 16.5%, respectively, ranking as the top two variables, reflecting the fact that precipitation of driest month (bio14), min temperature of coldest month (bio6), and precipitation of warmest quarter (bio18) are the key factors affecting the distribution of *C. hystrix* Miq.; the min temperature of coldest month (bio6) is dominant among the environmental factors.

**Figure 2:**
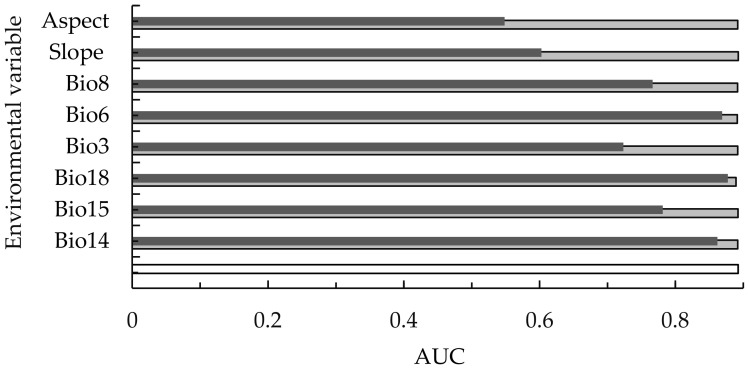
Jackknife test of MaxEnt model.

Figure 2 shows the evaluation results of the jackknife method. It can be seen that when there is only precipitation of warmest quarter (bio18) variables, the AUC value is the highest (0.8772) among the variables, followed by the min temperature of coldest month (bio6) and precipitation of driest month (bio14), indicating that precipitation of warmest quarter (bio18), min temperature of coldest month (bio6), and precipitation of driest month (bio14) play an important role in the distribution of *C. hystrix* Miq.

Overall, the order of importance of environmental variables varied in different evaluation methods. However, Table 1 and Figure 2 show that the min temperature of coldest month (bio6), precipitation of driest month (bio14), and precipitation of warmest quarter (bio18) were the three leading factors affecting the potentially suitable cultivation regions of *C. hystrix* Miq.

### 2.3. Potentially Suitable Castanopsis hystrix Miq. Cultivation Regions under Present Climatic Conditions

**Figure 3:**
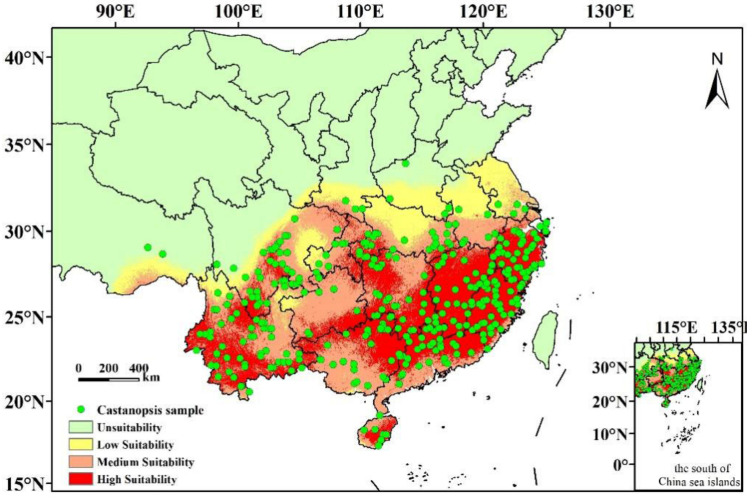
Potentially suitable cultivations regions of *C. hystrix* Miq. in China under present climatic conditions.

Figure 3 shows potentially suitable cultivation regions of *C. hystrix* Miq. under the present climate conditions predicted by the model. These modeling results were consistent with the actual pattern, and the horizontal distribution range is 18°–34° N, 89°–122° E. The natural discontinuity classification method was used to classify the distribution regions of *C. hystrix* Miq. according to suitability into four grades: high-suitability regions (0.47–0.86), medium-suitability regions (0.29–0.47), low-suitability regions (0.10–0.29), and unsuitable regions (0–0.10). The total suitability area is approximately 261.95 × 10^4^ km^2^ (Table 2), accounting for about 27.29% of the Chinese land area, mainly distributed in Guangdong, Guangxi, Hainan, and Yunnan in the south of China; Hubei, Hunan, Sichuan, Chongqing, and Guizhou in central, south, and southwest China; and Anhui, Zhejiang, Jiangxi, Fujian, and Jiangsu in east China. Additionally, Tibet, Gansu, Henan, and Shaanxi also have small potentially suitable cultivation regions.

The high-suitability area of *C. hystrix* Miq. in China is 101.78 × 10^4^ km^2^ (Table 2), accounting for 38.86% of the total suitability distribution area and 10.60% of the total Chinese land area. The areas were mainly distributed in ten provinces and one autonomous region in China, including Yunnan, Guangdong, Fujian, Zhejiang, Jiangxi, Hunan, Guizhou, Sichuan, Chongqing, Hainan, and Guangxi. The medium-suitability area is 102.10 × 10^4^ km^2^ (Table 2), accounting for 39.32% of the total suitability distribution area and 10.64% of the total Chinese land area. The medium distribution regions are closely linked to the high-suitability regions of *C. hystrix* Miq. and are surrounded by high-suitability regions. The low-suitability area is 57.17 × 10^4^ km^2^ (Table 2), accounting for 21.82% of the total suitability distribution area and 5.95% of the total Chinese land area. The areas were distributed in strips and were located at the outermost edge of the total suitability distribution regions of *C. hystrix* Miq., mainly Yunnan, Sichuan, Gansu, Shanxi, Hubei, Henan, Anhui, Jangsu Province, and the Tibet Autonomous Region. The results revealed that the potentially suitable distribution regions for *C. hystrix* Miq. were relatively concentrated, mainly distributed in central and southern China, and more than half of the suitable regions were moderately and highly suitable cultivation regions.

### 2.4. Spatial Distribution Pattern Change of Castanopsis hystrix Miq. under Future Climate Scenarios

**Figure 4:**
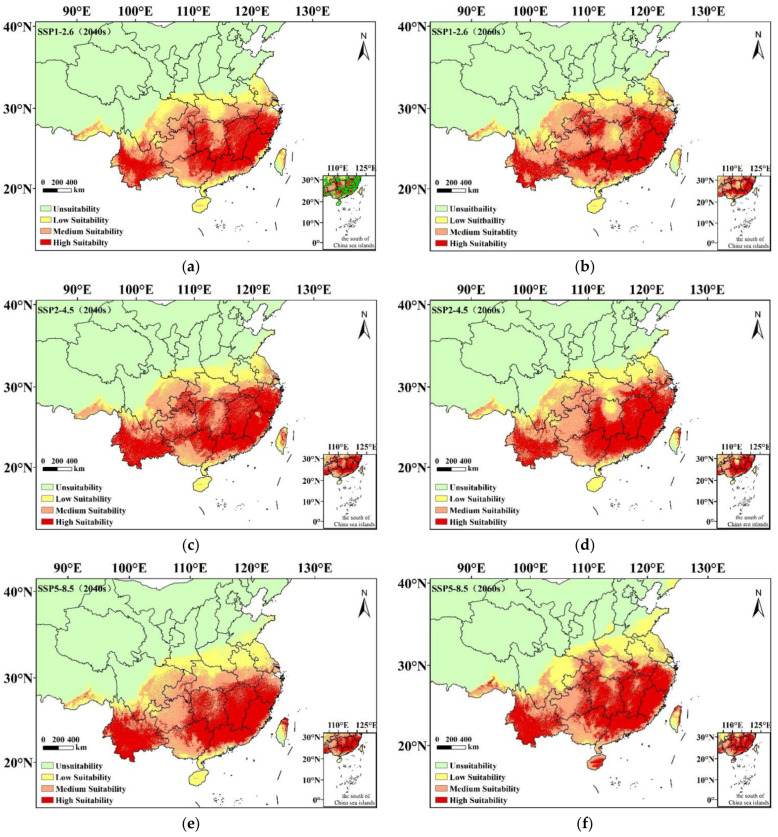
Potentially suitable cultivations regions of *C. hystrix* Miq. in China under future climatic conditions: (**a**). ssp1-2.6 (2040); (**b**). ssp1-1.6 (2040); (**c**). ssp2-4.5 (2040); (**d**). ssp2-4.5 (2060); (**e**). ssp5-8.5 (2040); (**f**). ssp5-8.5 (2060).

**Figure 5:**
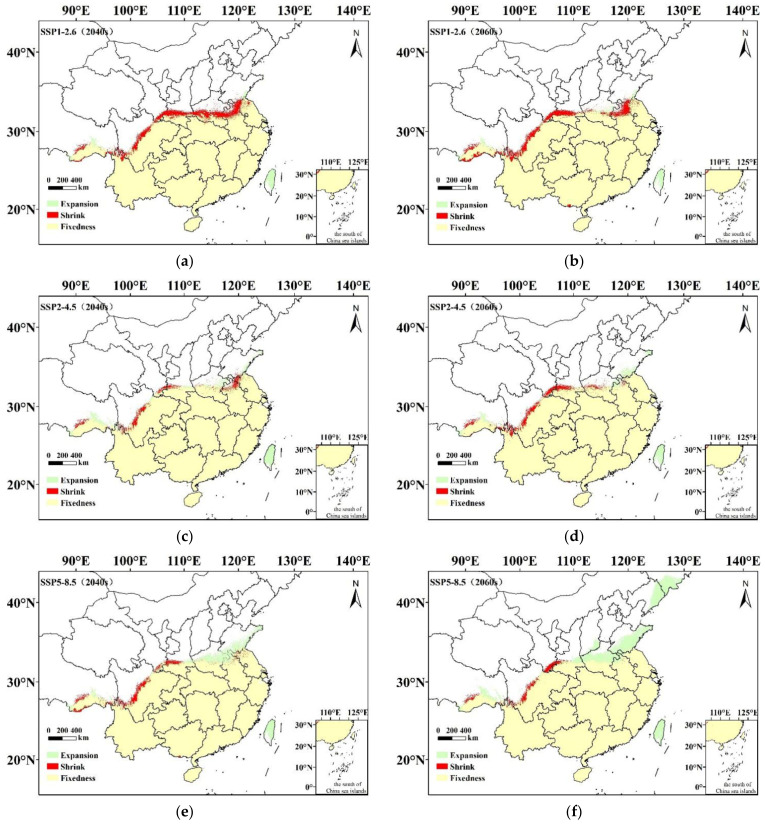
Spatial pattern change of suitable distribution for *C. hystrix* Miq. under future climatic conditions: (**a**). ssp1-2.6 (2040); (**b**). ssp1-1.6 (2040); (**c**). ssp2-4.5 (2040); (**d**). ssp2-4.5 (2060); (**e**). ssp5-8.5 (2040); (**f**). ssp5-8.5 (2060).

MaxEnt model simulations of the potential suitability distributions of *C. hystrix* Miq. in the 2040s and 2060s varied slightly, depending on the climate scenario. Compared with the prediction results under the present climatic conditions, most of the distribution regions of *C. hystrix* Miq. were retained. However, climate change could affect the spatial pattern and area of suitable cultivation regions (Figure 3, Figure 4 and Figure 5).

Through a comparative analysis of the future with the present suitable cultivation regions of *C. hystrix* Miq., it was found that under the ssp1-2.6 scenarios, the potential suitability distribution ranges of *C. hystrix* Miq. in western China tend to migrate to low latitudes, while the *C. hystrix* Miq. in eastern China tend to migrate to low latitudes and expand to Taiwan (Figure 5). Under the ssp2-2.4 scenarios, except for the added potentially suitable cultivation region in Taiwan, other migration changes were not obvious (Figure 5). For the ssp5-8.5 scenarios, the potential suitability distribution ranges of *C. hystrix* Miq. in the west continued to shrink to low latitudes, while *C. hystrix* Miq. in eastern China expanded to high latitudes (Henan, Shandong, Liaoning, Jilin) and Taiwan (Figure 5). The high-suitability regions of *C. hystrix* Miq. have a tendency to migrate to high latitudes (Figure 3 and Figure 4). There was no obvious migration change in the northern margin of medium-suitability regions, while the southern margin appeared to migrate to high latitudes (Figure 3 and Figure 4). A small number of low-suitability regions occurred in low latitudes of southern China, distributed in Hainan, Guangdong, Guangxi (Figure 3 and Figure 4).

With climate change, the potentially suitable cultivation area for *C. hystrix* Miq. showed a change compared to the present distribution area. Under the ssp1-2.6 scenario, the total potentially suitable cultivation area of *C. hystrix* Miq. decreased by 4.82% and 3.66% in the 2040s and 2060s, respectively (Table 2). The area with high suitability decreased considerably, whereas the area with low suitability increased slightly. The area of medium suitability decreased by 4.72% in the 2040s and increased by 2.85% in the 2060s (Table 2). For the ssp2-4.5, the total potentially suitable cultivation area of *C. hystrix* Miq. did not significantly change. It will increase by 0.48% in the 2040s and decrease by 0.71% in the 2060s. However, the area of medium suitability decreased significantly, whereas that of low suitability increased slightly. The area of high suitability increased by 0.74% in the 2040s and decreased by 9.30% in the 2060s (Table 2). In the ssp5-8.5 scenario, the total distribution area of *C. hystrix* Miq. increased by 1.78% and 10.18% in the 2040s and the 2060s, respectively. The area of medium suitability decreased, while the area of low suitability increased in the next two periods (the 2040s and 2060s). The highly suitable area decreased by 3.86% in the 2040s and increased by 5.62% in the 2060s (Table 2). In general, with climate change, a potentially suitable cultivation area for *C. hystrix* Miq. is the largest under the ssp5-8.5 scenario, 266.61 × 10^4^ km^2^ and 288.61 × 10^4^ km^2^, respectively, in the 2040s and 2060s, followed by ssp2-4.5 and ssp1-2.6.

## 3. Discussion

### 3.1. Reliability of Model Simulation Results

The species distribution data collected in this study were combined with the long-term field survey data of the project team, research literature on CNKI, and various plant digital archive platforms. Information on *C. hystrix* Miq. distribution was comprehensive and accurate, and the sample size was large, so the MaxEnt model was particularly accurate in predicting and estimating the potentially suitable distribution. In this study, eight variables with a large contribution rate were reserved through pre-modeling, which avoided over-fitting of the model due to the high correlation among variables. The AUC value is recognized as the best measure of the model prediction accuracy [19]. The AUC value simulated by the model was in the range of [0.9, 1] in this study; therefore, the model had high precision and an excellent prediction effect. Under the present climatic conditions, the potentially suitable cultivation regions for *C. hystrix* Miq. in China basically covers the present geographical distribution, which fully shows that the MaxEnt model is highly reliable in predicting the potential distribution of *C. hystrix* Miq. under future climatic conditions.

### 3.2. Main Environmental Variables Affecting the Species Distribution of Castanopsis hystrix Miq.

The distribution characteristics of plant populations are the comprehensive result of biological and abiotic factors, as well as the final embodiment of plant responses to the environment [19]. Climate is an important factor that affects species distribution [29]. The response of species distribution to climate is derived from their own physiological characteristics. Our study identified that the min temperature of coldest month (bio6), precipitation of driest month (bio14), and precipitation of warmest quarter (bio18) were the main bioclimatic variables affecting the species distribution of *C. hystrix* Miq. Among the three bioclimatic variables, two were related to precipitation, and one was related to temperature. This is consistent with the physiological characteristics of *C. hystrix* Miq, which is more suited to regions with warmth and humidity and is not resistant to drought [30]. Temperature is one of the most important factors that affect plant growth, development, reproduction, morphology, quantity, and distribution. It restricts the growth and development of plants and all physiological and biochemical changes in the body. Precipitation is also affected by temperature and can decrease with climate change, thereby enhancing drought stress and reducing soil moisture. Thus, drought and temperature instability could affect the distribution of *C. hystrix* Miq., inducing shifts, reductions, and expansion of distribution ranges and eventually changing the suitability distribution pattern. The main three bioclimatic variables (bio6, bio14, and bio18) emphasize “the coldest”, “the driest”, and “the hottest”, respectively, which reflect the temperature and rainfall level under extreme conditions; this result emphasized that the extreme climate in different regions significantly affects the survival and distribution of *C. hystrix* Miq. In addition, the distribution of species is inevitably affected by other abiotic factors, such as light, air, and soil, as well as biological factors, such as human factors and the influence between species [31]. The environmental variables selected in this study could not fully represent the geographical distribution factors affecting *C. hystrix* Miq. Therefore, all types of biological and non-biological factors can be considered in the model in future research, which also shows an important direction for future model development [32].

### 3.3. Potentially Suitable Cultivation Regions of Castanopsis hystrix Miq. under Present Climatic Conditions

The results of this study showed that the potentially suitable distribution regions of *C. hystrix* Miq. are relatively concentrated and mainly distributed in central and southern China, and the horizontal distribution range is 18°–39° N, 91°–122° E. The central and southern regions of China have a tropical monsoon climate and subtropical monsoon climate. The tropical monsoon climate is hot all year round, annual average temperature is above 22 °C, coldest month is basically above 18 °C, dry and rainy season is obvious, precipitation is concentrated in the rainy season; the precipitation is large, monsoon is significant, and the tropical cyclone is prevalent. The summer subtropical monsoon climate has high temperatures and is rainy, winter is mild and less rainy and rich in thermal resources. It can be seen that the results predicted by MaxEnt model were consistent with the habitat suitability of *C. hystrix* Miq. to climate and geographical environments. The total suitability area of *C. hystrix* Miq. accounted for about 27.29% of the Chinese land area; the high-suitability area accounted for 38.86% of the total suitability distribution area, and the medium suitability area accounted for 39.32% of the total suitability distribution area. These results revealed that the potentially suitable cultivation regions of *C. hystrix* Miq. are relatively concentrated in China and that they have certain limitations.

### 3.4. Spatial Distribution Pattern of Castanopsis hystrix Miq. under Climate Change

This study adopted a new scenario framework composed of shared socioeconomic pathways (SSPs) and representative concentration pathways (RCPs) proposed by the Intergovernmental Panel on Climate Change (IPCC), combined with the actual social and economic situation of China, to study the effects of three climate scenarios (ssp1-2.6, ssp2-4.5, and ssp5-8.5) on the potentially suitable distribution and spatial pattern migration of *C. hystrix* Miq. As an important part of the new generation of climate change scenarios, SSPs describe different tracks of future socioeconomic system development and reflect the relationship between the development model of the socioeconomic system and climate change risk [33,34]. Compared with the previous single climate scenario model, the current new scenario framework considers the future population, economy, technology, and policy, compensates for the uncertainty of socio-economic activities in previous climate scenario studies, and provides a more diversified emission scenario of air pollutants for a more accurate prediction of the changes in the natural environment and species distribution caused by future climate change [35].

Through the research results under future climatic conditions, we found that most of the potentially suitable cultivation regions for *C. hystrix* Miq. still remain and are still highly concentrated in the central and southern regions of China, indicating that *C. hystrix* Miq. has certain adaptability to climate change. However, climate change inevitably affects species suitability. Different climate scenarios in different periods, migration directions, and area of *C. hystrix* Miq. suitable regions were different. Under the climate scenarios of ssp1-2.6, the range of suitable regions for *C. hystrix* Miq. shows the trend of migration to low latitudes. This may be because under the social and economic path of sustainable development (SSP1), the global CO_2_ emissions have dropped significantly, the global warming process is slowing down, and temperatures and precipitation in some areas have changed significantly due to the strengthening and advancing monsoon and airflow, resulting in the climate environment no longer being suitable for the survival of *C. hystrix* Miq. [1,36,37]. Under climate warming scenarios, summer precipitation in central and western China would decrease significantly [38], which is also probably the reason for the suitable regions for *C. hystrix* Miq. migrating to the south. Under the ssp2-4.5 climate scenario, the spatial pattern of cultivation regions change was not significant, possibly because the intermediate development path (SSP2) is where socio-economic factors follow their historical trend and CO_2_ emissions hover around current levels before the middle of the century [39]. Under the ssp5-8.5 climate scenario, *C. hystrix* Miq. tends to expand to the northeast region at high latitudes. SSP5 refers to the rapid growth of the global economy driven by the exploitation of fossil fuels and energy-intensive lifestyles; by 2050, the level of CO_2_ emissions will roughly double compared with the current level [36,37,38,39]. In this climate scenario, the potentially suitable distribution regions for *C. hystrix* Miq. expand to high latitudes. With the increase in greenhouse gas emissions and average annual temperature, the increase of suitable distribution range in northern China is greater than that in southern China [39]. Thus, the newly added potentially suitable distribution regions were distributed in Henan, Shandong, Liaoning, and Jilin provinces. Numerous studies have also confirmed the migration of many other species to high latitudes in response to climate change [40,41,42]. In addition, climate warming would lead to an increase in precipitation intensity in the middle and high latitudes of the Northern Hemisphere and in the number of drought days in the middle and low latitudes [4], which is another reason for *C. hystrix* Miq. distribution regions migrating from low latitudes (Sichuan and Gansu) to high latitudes (Liaoning and Jilin). This migration is consistent with the growth habit of *C. hystrix* Miq., which includes preference for warmth and humidity.

The migration and expansion of the potentially suitable cultivation regions of *C. hystrix* Miq further led to changes in the distribution area. By 2040 and 2060, compared with other climatic conditions, the distribution area of *C. hystrix* Miq. under the ssp5-8.5 climate scenario were the largest, 266.61 × 10^4^ km^2^ and 288.61 × 10^4^ km^2^, respectively, followed by ssp2-4.5 and ssp1-2.6, which reflects the fact that climate change under the socio-economic path of high greenhouse gas emissions gives *C. hystrix* Miq. a wider range of suitable areas. According to the statistical results, the areas of medium and high-suitability regions have reduction phenomena under different climatic conditions, which indicates that climate warming still has a certain negative impact on the cultivation area of *C. hystrix* Miq. This may be because climate warming in recent years has led to frequent extreme high-temperature weather in various regions across the country, resulting in high temperature, low rainfall, increased arid areas, and poor growth quality of *C. hystrix* Miq; thus, the distribution of *C. hystrix* Miq. in medium and high-suitability areas was further affected [4,43].

## 4. Materials and Methods

### 4.1. Species Distribution Data

**Figure 6:**
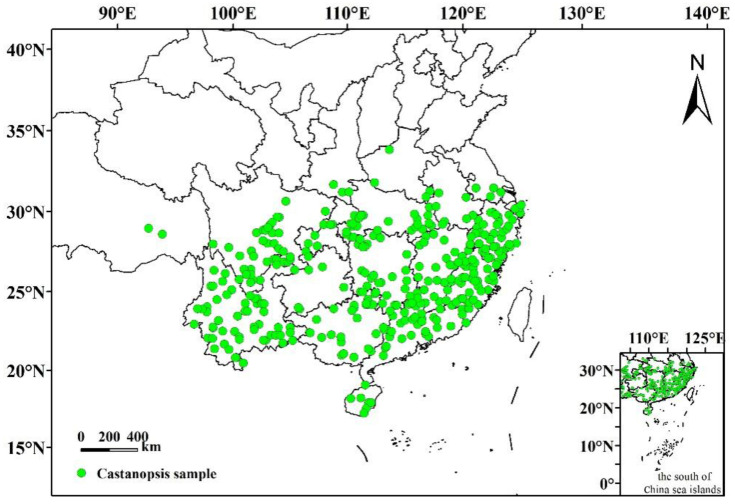
Sample distribution of *C. hystrix* Miq. in China.

Distribution data for *C. hystrix* Miq. were obtained from data platforms such as the Teaching Specimen Resource Sharing Platform (http://mnh.scu.edu.cn, accessed on 5 July 2022), Flora of China (www.iplant.cn/frps, accessed on 15 July 2022), the Global Biodiversity Information Network (http://www.gbif.org, accessed on 5 July 2022), the National Specimen Resource Sharing Platform (http://www.nsii.org.cn, accessed on 15 July 2022), the China Digital Herbarium (http://www.cvh.ac.cn, accessed on 15 July 2022), and field-investigation-related materials. In total, 1063 records of the distribution points were collected in China. The geographical coordinates of the distribution data were determined using the GAODE picker (https://lbs.amap.com/console/show/picker, accessed on 15 July 2022), and repeated records and uncertain distribution information were removed through Excel screening. Finally, 360 pieces of effective distribution data for *C. hystrix* Miq. were obtained from China (Figure 6). ArcGIS and Excel were used to process the data and the data were saved in (.csv) format. The converted file was used as the input to MaxEnt.

### 4.2. Environmental Data

A total of 22 initial environmental variables were used in this study, including 19 bioclimatic variables and the geographical variables (Table 3). The present climate data were retrieved by interpolating with anusplin4.36 software after being statistically sorted by the China Meteorological Data Network (http://data.cma.cn/, accessed on 26 July 2022) China monthly climate data set (1971–2021).

The IPCC had proposed a new scenario framework composed of socio-economic scenarios and climate scenarios in 2010 [35,36]. Among them, socio-economic scenarios are represented by Shared Socioeconomic Pathways (including the SSP1-sustainable path, SSP2-intermediate, SSP3-regional competition path, SSP4-unbalanced path, SSP5-fossil fuel-based development path). Climate scenarios are represented by Representative Concentration Pathways (including RCP2.6, RCP4.5, RCP8.5). SSP1-2.6, for example, indicates that under the sustainable shared socio-economic path, some climate mitigation/adaptation policies are implemented to achieve the 2.6 radiative forcing target. In this study, ssp1-2.6, ssp2-4.5, and ssp5-8.5 emission scenarios were chosen as future climate data, which were retrieved from the WorldClim Database (https://worldclim.org, accessed on 26 July 2022). Three geographical variables were retrieved from the Geospatial Data Cloud (http://www.scloud.cn, accessed on 26 July 2022). Twenty-two initial environment variables were uniformly processed by ArcGIS software and saved as (.asc) format.

Since environmental variables have drawbacks associated with multiple collinearity and dimensionality, it was difficult to build and interpret a model that considered all the environmental variables or determined which of them were relevant [44]. Therefore, to improve the accuracy of the model, MaxEnt was used to pre-model 22 variables to obtain the contribution rate of all variables, and SPSS software was used to analyze their correlation. In combination with the contribution rate of the environmental variables, all variables with a correlation of less than 0.75 were retained, and only variables with a high contribution rate were retained for variables with correlation ≥ 0.75. Through comparative analysis, six bioclimatic variables and two geographical variables were chosen as the input data for the environmental variables of the MaxEnt model (Table 3).

### 4.3. MaxEnt Model Construction and Operation Method

The MaxEnt model was constructed using the filtered and processed 360 species distribution data and eight environmental variables. The specific model parameters were set as follows: the bootstrap operation method was selected to run the model 10 times; the “random seed” was set to make each run use 75% of the random samples in the distribution data as the training set, and the remaining 25% of the samples as the test set. The “create response curve“ function was used to create the response curve of each climate variable to obtain the relationship between distribution probability and climate factors. The “Make pictures of predictions” function was selected to draw the potential distribution prediction map. The “Do jackknife to measure variable importance” function was selected, and the jackknife method was used to test all climate variables to evaluate their importance. The model takes the average value of 10 simulation results as the final output saved in Logistic format.

### 4.4. Data Analysis

#### 4.4.1. Model Prediction Accuracy Evaluation

In the MaxEnt model, the receiver operating characteristic (ROC) curve was used to evaluate the accuracy of the model, and the true positive rate and false positive rate were taken as the horizontal and vertical coordinates, respectively [29,30,31]. The area surrounded by the ROC curve and abscissa, namely the area under the curve (AUC), is often used as an indicator to evaluate the accuracy of the model because it is less affected by sample size and threshold [29,30,31]. The range of the AUC values was [0, 1]. Different AUC values indicate different model accuracies: poor [0.5, 0.6], fair [0.6, 0.7], good [0.7, 0.8], very good [0.8, 0.9], and excellent [0.9, 1].

#### 4.4.2. Importance Assessment of Environmental Variables

The MaxEnt model has two methods to evaluate the importance of environmental variables for the distribution prediction of *C. hystrix* Miq.: the percent contribution and permutation importance assessment method, and the jackknife test method [45]. The percentage contribution rate is the contribution value of each variable to the geographical distribution of vegetation given by the MaxEnt model in the training process, and permutation importance refers to the reduction degree of the AUC value obtained from the model simulation results after randomly replacing the climate variables of the training sample points. The greater the reduction value, the more dependent is the model on this variable. The second method is the jackknife test method, which excludes one or more variables in each turn and then analyzes the importance of a single variable in establishing the distribution model. When the AUC value of “only this variable” is high, it indicates that this environmental variable occupies a dominant position in the model prediction and has a great impact on the prediction results of species distribution. “Except for this variable” refers to the removal of a certain variable and using the remaining variables for modeling. A low AUC value indicates that the removed environmental variable contains key information that is important for species distribution prediction. “All variables” means to use all variables to predict the model [46].

#### 4.4.3. Classification of *Castanopsis hystrix* Miq. Potentially Suitable Cultivation Regions

Forecast results (.asc) were imported from the MaxEnt model into ArcGIS and converted into a grid data format (.tif). Then, the natural discontinuity classification method was used to reclassify the potentially suitable cultivation regions of *C. hystrix* Miq. into four grades: high-suitability regions, medium-suitability regions, low-suitability regions, and unsuitability regions. Finally, the current and future potentially suitable distribution pattern map of *C. hystrix* Miq. was generated using ArcGIS 10.2.

#### 4.4.4. Spatial Pattern Change of *Castanopsis hystrix* Miq. under Future Climate Scenarios

We compared and analyzed the potentially suitable distribution pattern of *C. hystrix* Miq. under different climate scenarios in different periods and graphed the spatial change trend map of suitable regions of *C. hystrix* Miq. The potentially suitable area of *C. hystrix* Miq. was calculated under different climate conditions and suitable levels to explore the spatial pattern change law of *C. hystrix* Miq. for each period under a climate scenario.

## 5. Conclusions

This study aimed to explore the habitat suitability of *C. hystrix* Miq. in China and its responses to climate change. The MaxEnt model was used to predict the potentially suitable distribution of *C. hystrix* Miq. under present and future climatic conditions. The simulation indicated that the temperature and precipitation conditions in the planting regions should be considered; furthermore, the temperature and precipitation conditions under extreme conditions should be emphatically analyzed when introducing and cultivating *C. hystrix* Miq. in a large area. A map of potentially suitable cultivation regions under the present climatic conditions can assist in the development of protection and restoration strategies. According to these results, the highly suitable cultivation regions for *C. hystrix* Miq. can be used as a breeding base for its excellent germplasm resources and is the best region for continued cultivation, popularization, and development of the *C. hystrix* Miq. industry to maximize its multi-benefit value. Medium-suitable cultivation regions for *C. hystrix* Miq. should be given attention, adhering to the principle of “suitable place to plant suitable trees,” planting, and popularizing *C. hystrix* Miq. scientifically, reasonably, and selectively. However, the low-suitability cultivation regions and unsuitable cultivation regions of *C. hystrix* Miq., should be reduced or avoided to prevent wasting human, material, and financial resources caused by blind introduction. A map of the spatial distribution of *C. hystrix* Miq. under future climatic conditions will help us to enact conservation strategies in advance for these regions to minimize the potential impacts of climate change. Our study identified that under the climate conditions of ssp1-2.6 and ssp2-4.5, the introduction and cultivation of *C. hystrix* Miq. should still focus on the southern region of high latitudes and appropriately reduce the planting area and distribution range at low latitudes. Its cultivation can be appropriately increased and expanded to the northeast of high-latitude areas by a small margin, under ssp5-8.5 climate conditions, on the premise of ensuring the normal growth of *C. hystrix* Miq.

Our study focused on the habitat suitability of vegetation and its response to global climatic change. The results mentioned above could aid in understanding the habitat suitability of *C. hystrix* Miq. and enacting a resource conservation policy to minimize the impact of climate change.

## Figures and Tables

**Table 1:** Contribution rate of each environmental factor in MaxEnt mode.

Variable Code	Describe	Contribution Rate (%)	Permutation Importance(%)
Bio14	Precipitation of driest month	55.4	5.4
Bio6	Min temperature of coldest month	31.9	61.9
Bio18	Precipitation of warmest quarter	2.9	16.5
Bio3	Isothermality	2.7	2.2
Slope	—	2.6	2.3
Bio8	Mean temperature of wettest quarter	2.2	6.3
Aspect	—	1.3	2.6
Bio15	SD of humidity seasonality	1	2.7

**Table 2:** Potential suitable area of *C. hystrix* Miq. under different climate change scenarios (×10^4^ km^2^).

Climatic Conditions		High Suitability	Medium Suitability	Low Suitability	Unsuitability
Current climate		101.78	102.10	57.17	698.05
SSP126	2040s	84.79	98.14	66.40	710.68
	2060s	86.71	105.94	59.72	707.64
SSP245	2040s	102.53	99.79	60.90	696.79
	2060s	92.31	100.89	66.88	699.92
SSP585	2040s	97.85	96.63	72.13	693.39
	2060s	107.50	98.67	82.45	671.39

**Table 3:** Description of climate data variables.

Describe	Unit	Choose	Describe	Unit	Choose
Mean annual temperature (Bio1)	°C		Annual precipitation (Bio12)	mm	
Mean diurnal range (Bio2)	°C		Precipitation of wettest month (Bio13)	mm	
Isothermality (Bio3)	—	P	Precipitation of driest month (Bio14)	mm	P
SD of temperature seasonality (Bio4)	°C		SD of humidity seasonality (Bio15)	—	P
Max temperature of warmest month (Bio5)	°C		Precipitation of wettest quarter (Bio16)	mm	
Min temperature of coldest month (Bio6)	°C	P	Precipitation of driest quarter (Bio17)	mm	
Temperature annual range (Bio7)	°C		Precipitation of warmest quarter (Bio18)	mm	P
Mean temperature of wettest quarter (Bio8)	°C	P	Precipitation of coldest quarter (Bio19)	mm	
Mean temperature of driest quarter (Bio9)	°C		Dem	m	
Mean temperature of warmest quarter (Bio10)	°C		Aspect	—	P
Mean temperature of coldest quarter (Bio11)	°C		Slope	°	P

## Data Availability

Data from data platforms such as the Teaching Specimen Resource Sharing Platform (http://mnh.scu.edu.cn, accessed on 15 July 2022), Flora of China (www.iplant.cn/frps, accessed on 15 July 2022), Global Biodiversity Information Network (http://www.gbif.org, accessed on 15 July 2022), National Specimen Resource Sharing Platform (http://www.nsii.org.cn, accessed on 15 July 2022), China Digital Herbarium (http://www.cvh.ac.cn, accessed on 15 July 2022), the GAODE picker (https://lbs.amap.com/console/show/picker, accessed on 15 July 2022), the China Meteorological Data Network (http://data.cma.cn/, accessed on 26 July 2022), WorldClim Database (https://worldclim.org, accessed on 26 July 2022), the Geospatial Data Cloud (http://www.scloud.cn, accessed on 26 July 2022). All data referred to or generated in this study are included in tables or figures and are available upon request.
